# Faith Community Engagement to Mitigate COVID-19 Transmission Associated with Mass Gathering, Uman, Ukraine, September 2021

**DOI:** 10.3201/eid2813.220183

**Published:** 2022-12

**Authors:** Lauren Erickson-Mamane, Alina Kryshchuk, Olga Gvozdetska, Dmytro Rossovskyi, Aaron Glatt, David Katz, Zvi Gluck, Deena Butryn, Yonathan Gebru, Laura Guerra, Alyssa Masor, Kathleen Blaney, Christopher A. Papaharalambus, Ezra J. Barzilay, Avi J. Hakim

**Affiliations:** Centers for Disease Control and Prevention, Atlanta, Georgia, USA (L. Erickson-Mamane, D. Butryn, Y. Gebru, A.J. Hakim);; Public Health Center of the Ministry of Health of Ukraine, Kyiv, Ukraine (A. Kryshchuk, O. Gvozdetska, D. Rossovskyi);; Mount Sinai South Nassau, Oceanside, New York, USA (A. Glatt);; Icahn School of Medicine at Mount Sinai, New York, New York, USA (A. Glatt);; Rabbinical Alliance of America, Brooklyn, New York, USA (A. Glatt, D. Katz);; Amudim Community Resources, New York (Z. Gluck);; New York City Test and Trace Corps, New York (L. Guerra);; New York City Department of Health and Mental Hygiene, New York (A. Masor, K. Blaney);; US Centers for Disease Control and Prevention Ukraine, Kyiv (C.A. Papaharalambus, E.J. Barzilay)

**Keywords:** COVID-19, coronavirus disease, severe acute respiratory syndrome coronavirus 2, SARS-CoV-2, coronavirus, viruses, respiratory infections, transmission, faith community engagement, contact tracing, physical distancing, mass gathering, mitigation, pilgrimage, pilgrim, vaccination, zoonoses, Uman, Ukraine

## Abstract

Annually, ≈30,000 Hasidic and Orthodox Jews travel to Uman, Ukraine, during the Jewish New Year to pray at the burial place of the founder of the Breslov Hasidic movement. Many pilgrims come from the northeastern United States. The global health implications of this event were seen in 2019 when measles outbreaks in the United States and Israel were linked to the pilgrimage. The 2020 pilgrimage was cancelled as part of the COVID-19 travel restrictions imposed by the government of Ukraine. To prepare for the 2021 event, the National Public Health Institute, the Public Health Center of Ukraine, organized mitigation measures for pilgrims arriving in Uman, and the CDC COVID-19 International Task Force assisted with mitigation measures for pilgrims coming from the United States. We describe efforts to support COVID-19 mitigation measures before, during, and after this mass gathering and lessons learned for future mass gatherings during pandemics.

The World Health Organization (WHO) characterized the spread of SARS-CoV-2, the virus that causes COVID-19, as a pandemic in March 2020 (*1*). At the onset of the pandemic, WHO recognized transmission risks during gatherings and subsequently issued guidance and policy documents for gatherings during the COVID-19 pandemic (*2*). Mass gatherings are defined by WHO as events involving large numbers of attendees at a specific location, for a specific purpose, over a specific duration of time (*3*). Given the high density and mobility of participants, mass gatherings can be associated with increased transmission of SARS-CoV-2. These gatherings can create conditions conducive for SARS-CoV-2 transmission, given crowding, challenges with physical distancing, and prolonged and frequent contact among mass gathering participants. Therefore, the WHO recommended that, during the pandemic, gatherings “should not take place unless the basic precautionary measures to prevent and control infection are strictly applied and adhered to by all attendees” (*2*). These basic precautionary measures include physical distancing, regular handwashing, adherence to mask guidance issued by local health authorities, staying outdoors and avoiding crowding, and ensuring proper ventilation when indoors.

Orthodox Jewish communities in New York City (NYC), New York, USA, have been disproportionally affected by COVID-19. In the early fall of 2020, the incidence of COVID-19 in Orthodox Jewish neighborhoods was 4 times higher than the citywide average (*4*). As of April 2020, the Hasidic neighborhood of Borough Park in Brooklyn had the second-highest number of COVID-19 cases in NYC, and the predominantly Orthodox County of Rockland County, New York, experienced the second-highest number of COVID-19 cases per capita in the United States (*5*).

Each year, ≈30,000 Hasidic and other Orthodox Jews travel to Uman, a city that has 86,900 persons in the Cherkasy Region of central Ukraine, during the Jewish new year, Rosh Hashanah, and subsequent high holy days, as part of a pilgrimage to pray at the burial place of Rabbi Nachman, an 18th century luminary who founded the Breslov Hasidic movement (*6*). During the Uman pilgrimage, pilgrims gather in tight quarters when praying in synagogues, as well as when sleeping and eating. Thousands of pilgrims travel to Uman from the United States, particularly from NYC (Amudim Community Resources, https://amudim.org). The global health implications of this event were seen in 2019 when measles outbreaks in the United States and Israel were linked to the pilgrimage (*7**,**8*). Cases of infection with SARS-CoV-2 in Ukraine were reported in March 2020 (*9*).

As of September 6, 2021, the beginning of Rosh Hashanah, 2,578,394 SARS-CoV-2 cases and 59,523 deaths had been reported in Ukraine (*10*). As part of the travel restrictions put in place by the government of Ukraine to prevent the spread of COVID-19, international travelers were not allowed to enter the country for the Uman pilgrimage in 2020 (*11*). Travel restrictions were lifted in 2021, enabling international travelers to participate in the Rosh Hashanah pilgrimage during September 6–8, 2021. Many pilgrims remained in Uman throughout the Jewish High Holidays that ended on September 29, 2021. Given the disruption of the 2020 pilgrimage, a larger number of pilgrims was expected for the 2021 Uman pilgrimage.

The Public Health Center of the Ministry of Health of Ukraine (UPHC), through the US Centers for Disease Control and Prevention (CDC) Ukraine Office, requested technical assistance from the CDC COVID-19 International Task Force in supporting mitigation efforts for this mass gathering, including specifically for pilgrims traveling from the United States. 

The purpose of this report was to describe COVID-19 mitigation measures for the 2021 Rosh Hashanah Pilgrimage to Uman, Ukraine; report the number of COVID-19 cases in NYC and Uman; and assess whether there were any signals of increased COVID-19 transmission in NYC linked to the Uman pilgrimage. The activities of the study, and the partnerships involved herein, exemplify the Supplement theme of Leveraging and Adapting Global Health Systems and Programs During the COVID-19 Pandemic. 

This activity was reviewed by CDC and determined to be nonresearch. It was conducted consistent with applicable federal law and CDC policy (see, e.g., 45 C.F.R. part 46, 21 C.F.R. part 56; 42 U.S.C. §241(d); 5 U.S.C. §552a; 44 U.S.C. §3501 et seq.). 

The CDC COVID-19 International Task Force engaged multiple groups, including influential American Hasidic leaders, to develop a comprehensive mitigation and communications strategy for the Uman pilgrimage targeting pilgrims in Ukraine and the United States. The project was coordinated by CDC in partnership with UPHC, the Rabbinical Alliance of America, Amudim, and the NYC Department of Health and Mental Hygiene (NYC DOHMH). CDC has been partnering with UPHC since its inception in 2017. During the COVID-19 pandemic, UPHC received funds from CDC to support faith-community engaged contact tracing and mitigation during the Rosh Hashanah pilgrimage. The Rabbinical Alliance of America and Amudim did not receive governmental funding for this study. 

Together, project partners developed a fact sheet based upon WHO and CDC COVID-19 guidelines, addressing the need for COVID-19 vaccinations before travel, and mitigation measures during the pilgrimage and upon return to the United States. The fact sheet was translated into Hebrew, Yiddish, and Ukrainian. Forward and backward translation in all languages was conducted in the United States and Ukraine by certified translators. Cognitive testing of the fact sheet was conducted with religious leaders and other community consultants to ensure cultural appropriateness.

Funded by the NYC DOHMH, the fact sheet was disseminated by full-page inserts in 5 major Orthodox publications (Hamodia, Flatbush Jewish Journal, Yated, Der Yid, and Di Tzeitung/News Report) in NYC for 5 days each during August 5‒September 3, 2021, reaching an estimated daily readership of 321,000 persons. The fact sheet was also sent out by various WhatsApp groups. Furthermore, a weekly YouTube show hosted by Rabbi Dr. Glatt, sponsored by Young Israel of Woodmere, New York and promoted by the Rabbinical Alliance of America and other national rabbinical organizations, regularly featured CDC mitigation guidance and reached over 6,000 viewers weekly. The Rabbinical Alliance of America also shared the fact sheet with its listservs, comprising >950 Orthodox rabbis and 1,500 additional congregational and community leaders.

In Uman, concerted COVID-19 mitigation efforts were made by UPHC (Table 1) and the US-based Orthodox Jewish faith-based organization, Amudim (Table 2). UPHC produced 2 videos aimed at the Hasidic Jewish community conveying recommendations for the safe celebration of Rosh Hashanah that were broadcast at the international airports in Kyiv and Lviv, large international airports used by most pilgrims traveling into Ukraine, and in hotels and refectories in Uman. A central billboard was also used on Pushkina Street, the main thoroughfare for the pilgrimage. “Safe Celebration of the Jewish High Holidays” web content was posted across the UPHC website; on the Visit Ukraine Web site, the principal tourist information portal for travelers to Ukraine and Ukrainians planning travel abroad; and through various social media outlets. Recognizing the need to immediately diagnose cases and isolate persons who had COVID-19, UPHC provided 50,000 CLINITEST Rapid COVID-19 antigen tests (Siemens, https://www.siemens.com) to Uman for use during the pilgrimage.

**Table 1:** COVID-19 mitigation measures implemented by UPHC and associated with mass gathering, Uman, Ukraine, September 2021*

Mitigation measure	Location	Quantity
Safe celebration of Rosh Hashanah video in English and Hebrew	International airports of Kyiv and Lviv, hotels and refectories in Uman, and central billboard on Pushkina Street, the main thoroughfare for the pilgrimage	2 videos: 1 in English with Hebrew subtitles, 1 in Hebrew with English subtitles
Safe celebration of Jewish high holidays web content	UPHC Web site, Visit Ukraine Web site (principle tourist information portal for travelers)	
Distribution of CDC-developed fact sheet	Rabbi Nachman of Breslov International Charitable Foundation and pilgrimage in Uma	19,000 fact sheets distributed
COVID-19 mitigation posters	Kyiv and Lviv airports, hotel lobbies, Red Cross tents, local synagogues, and Rabbi Nachman’s burial place	30 posters
COVID-19 hotline: Hebrew language option	Nation	1 national hotline
CLINITEST Rapid COVID-19 antigen tests† distributed	Uman	50,000
Hand sanitizer	Distributed to pilgrims in Uman	19,000
Disposable masks	Distributed to pilgrims in Uman	190,000

*CDC, Centers for Disease Control and Prevention; UPHC, National Public Health Center of the Ministry of Health of Ukraine. †Siemens (https://www.siemens.com).

**Table 2:** COVID-19 mitigation measures implemented by Amudim and associated with mass gathering, Uman, Ukraine, September 2021

Mitigation measure	Location	Quantity
COVID-19 mitigation banners and posters	Banners placed on the exterior of building on Pushkina Street and throughout dining and prayer areas	120 posters
COVID-19 mitigation cards	Distributed to pilgrims in Uman	250,000
Limiting capacity in the dining halls and at individual dining tables was limited to 50%	Uman	
Hand sanitizer and sanitizing hand wipes	Distributed to pilgrims in Uman	5,000 bottles of sanitizer, 100,000 wipes

Because the immediate goal was preventing the spread of COVID-19 during the Uman pilgrimage through multiple interventions in the United States and Ukraine, an evaluation of the interventions was not planned as part of the study. However, efforts were made to assess the effect of the study by comparing different data sources.

During the 2021 Uman pilgrimage, UPHC collaborated with the Uman branch of the Uman district of the Cherkasy Central Committee of the Ministry of Health to provide the number of PCR tests used and rapid antigen tests conducted in Uman, as well as the number of positive test results. UPHC also provided the number of COVID-19 cases among service workers in Uman during the pilgrimage and 2 weeks after the pilgrimage.

US Customs and Border Protection (USCBP) provided data regarding the number of travelers returning to NYC from Ukraine after Rosh Hashanah and other high holy days. CDC subsequently provided traveler data to the NYC Test and Trace Corps program (T2). Travelers were advised on quarantine and offered testing and vaccination resources.

COVID-19 incidence in Ukraine for the epidemiologic week starting September 6, 2021, was 6.3 cases/100,000 persons and increased to 10.4 cases/100,000 persons the next epidemiologic week (*10*). Pilgrims entering Ukraine were required to show a negative COVID-19 PCR test result for a test that was conducted no more than 72 hours before entering Ukraine. According to information received from the Head of the Situational Center of the Main Department of National Police in the Cherkasy region, 34,069 pilgrims came to Uman in 2021 to celebrate Rosh Hashanah, many of whom were US citizens. (Information was received on September 15, 2021, from the Head of the Situational Center of the Main Department of National Police in the Cherkasy region of Serhiy Kovalenko.) That center was created in Uman for the pilgrimage and included representatives of the State Emergency Service of Ukraine, the National Guard, the National Police, and the Border Guard Service. Information about the number of pilgrims was collected through various sources: the Border Guard Service, the International Charitable Rabbi Nachman Fund, and in the 7 points of entry to Uman (by the National Police). COVID-19 vaccination coverage among pilgrims is not known.

Although more pilgrims than usual were expected, given that international travelers were denied entry to Uman in 2020, official reports registered 34,069 pilgrims, a number similar to previous pilgrimages. Amudim reported less crowding in dining facilities than in previous years even if persons did not maintain 2 meters of physical distancing. For example, instead of having 14 persons at dining tables, table wardens ensured that only 6–8 persons used a table at a time. Temporary synagogues and areas of study were set up in Uman to decongest established synagogues that restricted prayers at 50% normal capacity.

During September 6–10, 2021, laboratories in Uman performed 13,267 PCR tests for symptomatic and asymptomatic persons and found 93 positive specimens (0.7% positivity), all among pilgrims. Rapid antigen tests (n = 3,467) were also performed, and none showed positive results. According to the Uman District Department of the Cherkasy Central Committee of the Ministry of Health, 11 additional positive COVID-19 PCR results were identified among pilgrims during predeparture screening at Kyiv Boryspil International Airport. As of September 21, 2021, no COVID-19 cases were registered among Rosh Hashanah service workers or the population in the pilgrimage zone for whom testing was readily available. The Situational Center of the Main Department of National Police in Cherkasy reported that as of September 22, 2021, there were 3,315 pilgrims remaining in the city of Uman. However, no additional testing data were provided during September 6–10, 2021.

The US government imposed COVID-19 mitigation measures for international travelers arriving in the United States, including mask mandates on all US airlines. All air passengers, including US citizens and fully vaccinated persons, were required to have a negative COVID-19 test result within 3 days before date of travel or documentation of COVID-19 recovery in the previous 3 months. A total of 9,936 international air passengers arrived in the United States from Ukraine the week after the pilgrimage, September 8–15, 2021. Most of those passengers were indirect arrivals with connections through airports in Europe. Slightly more than half (n = 5,219, 52.5%) of the total international air passengers from Ukraine arrived at John F. Kennedy (n = 3,661, 36.8%) and Newark (n = 1,558, 15.7%) airports as US First Ports of Entry (*12*). (Note that cited CBP data are US government–controlled information and, because of legal restrictions, may not be shared beyond provision of this manuscript without explicit written permission; written requests for information may be submitted to DHS-SPS-RFI@hq.dhs.gov). Given the potential for COVID-19 exposure during the Uman pilgrimage, T2 proactively reached out to 471 contactable travelers returning to NYC from Ukraine during September 8–15, 2021, the period after Rosh Hashanah, and an additional 404 contactable travelers returning to NYC from Ukraine during September 30–October 7, after the end of the high holy days, on the basis of lists provided by CDC using data supplied by USCBP. Travelers were called and given information about quarantine and offered resources on testing and vaccination. Passengers arriving during September 16–29 were not tracked by USCBP because stakeholders reported that the preponderance of pilgrims would return to the United States either after Rosh Hashanah or after the end of all high holy days.

In addition to the proactive call made to travelers, T2 performed case investigations on NYC residents who had positive laboratory-based or point-of-care SARS-CoV-2 test results, at which point contacts were elicited and details about recent travel were captured. During September 8‒October 8, 2021, T2 identified 15 persons who had COVID-19 and reported recent travel to Ukraine. These case-patients provided 22 contacts. T2 investigators observed lower than normal completion rates on case investigations and reluctance to respond to the question “have you traveled?” Although there was increased incidence of COVID-19 in 2 Hasidic neighborhoods in Brooklyn (Borough Park and Williamsburg) during September 2021, T2 determined the increase in Borough Park was unrelated to the pilgrimage because it began before the return of pilgrims; the cause for the increase in Williamsburg is unclear. We compiled COVID-19 incidence rates for these 2 communities (Table 3) (*13*).

**Table 3:** COVID-19 incidence rates associated with mass gathering, Uman, Ukraine, September 2021, in Brooklyn, New York, USA

Location	Beginning of epidemiologic week	Incidence, cases/100,000 persons
Borough Park, postal code 11219	Sep 6	79.93
	Sep 13	134.32
	Sep 20	164.29
	Sep 27	153.19
	Oct 4	167.62
	Oct 11	185.39
Williamsburg, postal code 11211	Sep 6	103.12
	Sep 13	131.79
	Sep 20	159.17
	Sep 27	134.35
	Oct 4	142.06
	Oct 11	155.32


The COVID-19 mitigation efforts for the Uman pilgrimage were a unique collaboration between the CDC COVID-19 International Task Force; Ukraine’s Public Health Center; the CDC Ukraine Office; the CDC COVID-19 State, Tribal, Local, and Territorial Task Force; Orthodox and Hasidic leaders in the United States; and the NYCDOHMH. These efforts highlight the opportunities to mitigate COVID-19 transmission associated with mass gathering events by focusing on mitigation before, during, and after an event. These efforts also highlight the critical need for early planning to coordinate the efforts and interests of diverse participants as it relates to mass gatherings.

Collaborations take time to develop, and cross-border collaborations can take even longer. We also learned that modified data systems might be needed to measure the effect of mitigation efforts during a mass gathering.

Ensuring that culturally appropriate and relevant communications materials were developed and disseminated by trusted entities was the cornerstone to programmatic success. Identifying and developing relationships with key Hasidic leaders in Uman and the United States was critical to mitigation efforts for the Uman pilgrimage. Planning started in February 2021 and required coordination across countries and jurisdictions. Creating culturally appropriate resources for diverse communities required extensive community consultation and piloting. Limited internet uptake among the target population required the of nontraditional communication channels, such as the use of WhatsApp groups, and printing communication materials for distribution. Partnerships enabled multiple communication touchpoints, including Orthodox Jewish print publications and hotlines, videos shown in airports and on major airlines, fact sheets, and COVID-19 hygiene kits and billboards. Mitigation measures focused on vaccination before travel, social distancing and mask wearing during the pilgrimage, and symptom monitoring, as wells as COVID-19 testing and contact tracing.

Future mass gatherings might consider supplementing routine data collection tools with tools specific to the mass gathering to better enable disaggregating test results between host community members and mass gathering participants. A registration system could also assist with active follow-up of mass gathering participants upon their departure and help identify COVID-19 cases associated with the mass gathering.

The positive outcomes of the mitigation efforts for the 2021 Uman pilgrimage were strengthening the partnership between CDC and the UPHC, the collaboration between CDC and the NYCDOHMH, and developing relationships and collaboration with Orthodox and Hasidic leaders in the greater NYC metropolitan area. This program underscored opportunities for future research for enhancing and targeting COVID-19 surveillance efforts to help identify where to focus mitigation efforts for future mass gatherings during pandemics.
